# Ciprofloxacin-Based Ionic Liquids Increase Mutation Frequency in *Escherichia coli*

**DOI:** 10.3390/antibiotics15060629

**Published:** 2026-06-22

**Authors:** Patrick Mikuni-Mester, Birgit Bromberger, Timea Dömök, Daniela Zetner, Laura Schleifer, Olga Makarova

**Affiliations:** 1 Centre for Food Science, University of Veterinary Medicine Vienna, Veterinaerplatz 1, 1210 Vienna, Austria; birgit.bromberger@vetmeduni.ac.at (B.B.);; 2 Centre for Veterinary Public Health and One Health, University of Veterinary Medicine Vienna, Veterinaerplatz 1, 1210 Vienna, Austria

**Keywords:** ionic liquids, *Escherichia coli*, mutation frequency, antibiotics, API-ILs, ciprofloxacin

## Abstract

**Background/Objectives:** Formulating antibiotics as active pharmaceutical ingredient ionic liquids (API-ILs) has been proposed as a strategy to help overcome antimicrobial resistance. However, the effects of API-ILs on bacterial mutation frequency, an increase of which is associated with a higher risk of resistance development, have not yet been assessed. Here, API-ILs based on the antibiotic ciprofloxacin were synthesized using five structurally different counter ions of varying biological activity - low ([Chol]^+^ and [EMMor]^+^), intermediate ([TMC_10_A]^+^) and high ([TMC_16_A]^+^ and [TC_8_MA]^+^) - and investigated in terms of their antimicrobial activity and mutation frequency in *Escherichia coli* MG1655. **Methods:** API-ILs were synthesized according to the CBILS© route. Conductivities and antimicrobial activity (determined by minimal inhibitory concentrations (MICs) and disk diffusion (DD) assays) of API-ILs as well as of individual API and ILs were measured, followed by mutation frequency assays. **Results:** Five novel ciprofloxacin-based API-ILs were synthesized. Overall, a lower dissociation of API-ILs compared to the respective ILs was observed, indicating presence of stable ion pairs in aqueous solution. All API-ILs retained the antimicrobial activity of ciprofloxacin. A higher mutation frequency (2.6–6.99-fold increase) was observed for API-ILs than for ciprofloxacin alone (1.71-fold increase), when compared to no treatment control, while ILs alone had no or a moderate impact (0.62–1.65-fold increase). **Conclusions:** Although it is possible to synthesize novel stable API-IL compounds with a high antimicrobial activity using ciprofloxacin and ILs of different structural classes, this can result in increased bacterial mutation frequencies. It is therefore crucial to improve our understanding of how API-ILs can be designed in a safer way.

## 1. Introduction

Ionic liquids (ILs)—organic salts with melting points below 100 °C—have recently attracted a lot of attention in the biomedical field as promising candidates for antimicrobial drug development due to their highly tunable nature and the ability to improve chemical and physical properties of pharmaceuticals. By modifying the structure of ILs, it became possible to overcome multiple problems associated with conventional solid state drugs, such as poor bioavailability, stability and polymorphism, as well as biological activity and environmental toxicity [1,2,3,4,5,6,7,8,9]. Indeed, their efficacy against bacterial pathogens has already been demonstrated individually (as IL cations/anions) and as part of active pharmaceutical ingredient ionic liquids (API-ILs) [2,5,10,11,12,13,14,15,16], including API-ILs based on ampicillin [17,18,19], nalidixic acid [20,21] and tetracycline [22]. For some of the ILs, the mode of action has been elucidated, which, depending on the chemical structure of the ion, involved disrupting cell membrane function, engaging with essential cellular elements such as proteins and DNA, inducing oxidative stress and disrupting metabolic functions [13,23]. The wide variety of cellular targets on the one hand might explain their effectiveness against antimicrobial-resistant (AMR) bacteria [13,17,24] but potentially could lead to an increased development of new resistances [20,25].

Spontaneous mutational events are one of the three major mechanisms that can drive antimicrobial resistance [26] and can be expressed through mutation rate (the rate at which spontaneous mutations arise per cell division) or mutation frequency (the number of mutants present in a population at a given time point). Both metrics reflect the same underlying mechanisms, although mutation rate is considered to be more accurate but also more difficult to measure than mutation frequency [27,28]. It has previously been shown that many antibiotics activate bacterial stress responses, leading to the expression of error-prone polymerases that result in increased mutation rates. Indeed, when treated with ampicillin, ciprofloxacin and kanamycin, bacteria show a 3-to-4-fold increase in mutation rate [29]. These increased mutation rates contribute to the increased standing genetic variation, on which selection can act, therefore creating the vicious cycle whereby antibiotic use may fuel antibiotic resistance. It is therefore important to determine at early stages of drug development whether a new antibiotic may increase the probability of mutational events in target bacterial species. Although mutation frequency provides an easy measure of mutagenicity, surprisingly, it has not yet been experimentally tested for ILs.

In this study, we investigated the mutation frequency of *Escherichia coli* exposed to API-ILs based on a popular antibiotic that is on the WHO list of essential medicines—ciprofloxacin (fluoroquinolones). Ciprofloxacin is known to increase the mutation rate of bacteria and previous studies already demonstrated that API-ILs with synergistic effects and improved bioavailability can be synthesized [30,31]. For this study, API-ILs with five different counter cations were synthesized. The five counter cations comprised two non-active, one medium active and two highly antimicrobially active structural motifs, which allows possible structure-dependent as well as activity-dependent effects if such effects exist. By also including “normal” chloride-based ILs of each counter cation, as well as the pure antibiotic, we also can determine if possible changes in mutation frequency are connected to the ILs *per se* or are a consequence of the API-IL. To the best of our knowledge, this is the first study investigating possible effects of ionic liquids or API-ILs on bacterial mutation frequencies. Information on potential mutagenicity of API-ILs is not only important for potential application as novel antibiotics but also for considering their increasing presence in natural environments [32,33].

## 2. Results and Discussion

### 2.1. Synthesis and Characterization of Novel API-ILs

**Figure 1:**
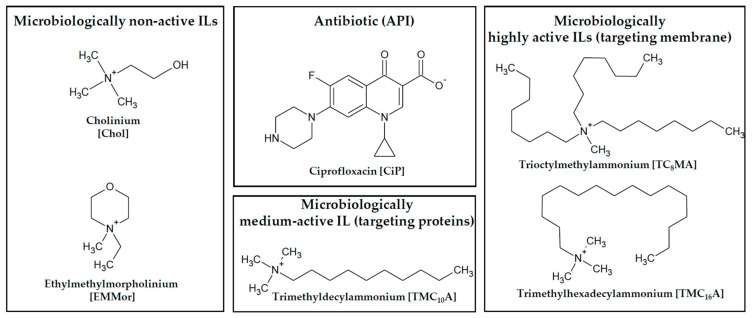
Schematic representation of the respective cations and anions of the 5 API-ILs investigated in this study.

In this study, novel API-ILs based on the antibiotic ciprofloxacin were synthesized using the CBILS© route, which is a completely halide and waste-free process producing high-purity ionic liquids [34]. We chose a set of five counter ions with varying antimicrobial activity to investigate possible synergistic effects of the new API-ILs in terms of antimicrobial activity and mutation frequency. The cations [Chol]^+^ and [EMMor]^+^ have a low antimicrobial activity, [TMC_10_A]^+^ has an intermediate activity, and both [TMC_16_A]^+^ and [TC_8_MA]^+^ are highly antimicrobial due to the presence of long or multiple alkyl side chains (Figure 1). Minimal inhibitory concentration (MIC) for each antibiotic, the respective chloride ILs of the cations and the API-ILs were determined (Table 1). To account for the differences in molecular weight of the API-ILs, in addition to the unit mg/L, which is usually used for antibiotics, the respective mM concentrations are also given.

To determine the degree of API-IL dissociation in aqueous solution, conductivity measurements for all compounds were performed and expressed as limiting molar conductivity (see Supplemental Tables S1 and S2 for full results). The degree of dissociation of API-ILs can support the identification of possible synergistic or sum effects of the respective cations and anions. Overall, a lower dissociation of the API-ILs compared to the respective chloride ILs was observed, indicating formation of stable ion pairs between the respective cation and anion.

For ciprofloxacin-based API-ILs, all MIC values were within the 2-fold resolution limit of the microbroth dilution method indicating a similar activity compared to pure ciprofloxacin. As the MIC values for the five API-ILs are significantly lower compared to the respective chloride ILs, no indication of an inactivation of the antibiotic during synthesis was found.

In addition to the microbroth dilution assay, the antimicrobial activity of the newly synthesized API-ILs and respective chloride ILs was tested by the disk diffusion test. For each compound, three different concentrations were tested, and representative results are listed in Table 2 (for complete results see Supplemental Table S3). In the case of the chloride ILs, only for [TMC_10_A][Cl] a small inhibition zone was detectable at 50 mg/disk, which was the highest tested concentration of the API-ILs.

Only small differences between the five API-ILs were detectable, with [TC_8_MA][CiP] showing the smallest inhibition zone. However, inhibition zones for API-ILs were all smaller compared to pure ciprofloxacin. This effect can be due to the higher molecular weight of the API-ILs resulting in a lower amount of API molecules, but also slower diffusion rates of the API-IL or the [CiP]- anion within the agar. A similar effect has been reported previously and thus it is recommendable to combine different methods when determining API-IL activity [35,36]. Overall, disk diffusion and MIC results demonstrate that the antimicrobial activity of ciprofloxacin-based API-ILs is equal to that of pure ciprofloxacin.

In summary, the results of both the microbroth dilution assay and the disk diffusion assay confirm the successful synthesis of the five API-ILs based on ciprofloxacin. The API-ILs showed activity both in the microbroth dilution test as well as the disk diffusion test, with the latter revealing different diffusion rates of the API-ILs compared to the pure antibiotic. This effect could be due to the bigger size and increased hydrophobicity of the respective associated API-ILs anions, which would explain why the bulky [TC_8_MA]^+^-based API-IL demonstrated the smallest inhibition zones.

### 2.2. Mutagenesis of API-ILs

For the analysis of API-IL mutagenesis, the ½ MIC concentrations were used for each of the API-ILs, pure ciprofloxacin and the five chloride ILs (Table S4). This concentration was chosen based on our own preliminary and published data demonstrating that half MIC induced the highest increase in mutagenesis by ciprofloxacin [37] and the expectation that environmental contamination would likely be at the sub-MIC concentration due to the dilution effects.

**Figure 2:**
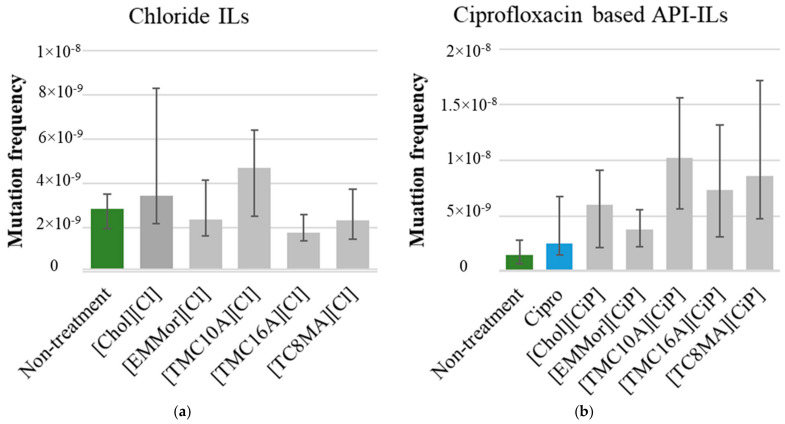
Changes in mutation frequency in *E. coli* MG1655 induced by chloride-based ionic liquids (**a**) and by ciprofloxacin-based API-ILs (**b**) at ½ MIC. Error bars represent 95% confidence interval for mutation frequency estimation by plating in rifampicin (100 mg/mL) and using the maximum likelihood method for data analysis. The non-treatment control in both figures is highlighted in green while the ciprofloxacin (cipro) control is highlighted in blue.

Figure 2 depicts the median mutation frequency for *E. coli* MG 1655 exposed to all substances shown (see Table S5). In good accordance with the literature, higher mutation frequencies were found for bacteria exposed to ciprofloxacin, although the increase of 1.71 was lower compared to previous studies in which 3- to 4-fold increases were detected [29]. The discrepancy can be due to differences in experimental conditions, such as different concentrations of the drugs and duration of treatment that have been shown to affect mutation rate [37]. For the chloride ILs, only in the case of [TMC_10_A][Cl] and [Chol][Cl] mutation frequencies were increased (1.65- and 1.21-fold, respectively), while for the other chloride ILs no impact on mutation frequency was found compared to the non-treatment controls (Figure 2a, Table S5). Therefore, a possible impact of the respective cations on mutation frequency changes in the cases of [EMMor][Cl], [TMC_16_A][Cl] and [TC_8_MA][Cl] can be excluded. It is important to point out that we did evaluate the possible mutagenesis of ILs *per se*, as only relatively low concentrations based on the respective API-ILs were tested. To the best of our knowledge, to date solely directed evolution experiments with the aim to increase ionic liquid tolerance (mostly for biomass applications) have been conducted but nothing is known regarding the general or structure-dependent mutagenicity of ionic liquids [38,39].

For ciprofloxacin-based API-ILs, not only were the observed mutation frequencies higher compared to the non-treatment control but also compared to ciprofloxacin alone. While for [EMMor][CiP] the mutation frequency was only slightly increased, for the other API-ILs, a 2–3-fold higher mutation frequency compared to ciprofloxacin could be found (Figure 2b). The apparent increased mutation frequency may be a result of the *de novo* mutagenesis by API-ILs, or, alternatively, a reflection of the preferential recovery or enrichment of pre-existing resistant mutants in response to the mutagenic effects of ciprofloxacin. Regardless of the underlying cause, given the fact that, due to the higher molecular weight, the molecular equivalent of ciprofloxacin is lower, a connection between the formulation as an API-IL and an increased mutation frequency is clear. As the respective chloride ILs did not lead to a similar increase at significantly higher concentrations, it is unlikely that the observed increased mutagenesis is a simple sum of the anion (in this case ciprofloxacin) and cation effects but rather a new intrinsic property of the API-IL. However, we also found clear differences between the API-ILs with non-antimicrobially active cations ([Chol][Cip] and [EMMor][Cip]) and the active ones ([TMC_10_A][Cip], [TMC_16_A][Cip] and [TC_8_MA][Cip]), demonstrating once more that the respective molecule structure can influence the outcome. As all of the three microbiologically active cations target or at least interact with bacterial membranes, an increased penetration of the API-IL or reduced efflux could be the underlying cause. It will be important in future studies to further increase the structural diversity of API-IL cations by including different head groups and side chain functionalizations as well as different antimicrobial modes of action [1,13]. The ultimate goal would be to develop quantitative structure–activity relationship (QSAR) models for mutation frequency prediction that can serve as an early warning system for mutagenicity of novel API-ILs, not only for procaryotic but also for eucaryotic cells.

## 3. Materials and Methods

### 3.1. Ionic Liquids and Other Chemical Substances

The five chloride-based ionic liquids used in this study were provided by Proionic GmbH (Grambach, Austria) with a nominal purity of >98%. All API-ILs based on ciprofloxacin were synthesized in our laboratory, according to the CBILS route (CBILS is a registered trademark of Proionic GmbH) [40,41]. Cationic precursors for API-IL synthesis were provided by Proionic GmbH (Grambach, Austria) with a nominal purity of >98% and ciprofloxacin was purchased from Sigma-Aldrich (Vienna, Austria). All investigated ILs and API-ILs are summarized in Table 1 and Figure 1.

### 3.2. Conductivity Measurements

The specific conductivities were measured using a Seven Compact Cond meter S230 (Mettler Toledo GmbH, Vienna, Austria). Initially, 10 mL of a 25 mM aqueous solution (ddH_2_O) of each compound was prepared and conductivity measurements were made at 25 °C. A known amount of ddH_2_O was added stepwise to the sample to obtain the required concentration (50 mM to 1.5 mM) and conductivity was measured after each addition (see Tables S1 and S2). The molar conductivity was calculated using the following equation: Λm = (k/c). The limiting molar conductivity Λ°m was determined graphically by plotting Λm vs. √C according to the Kohlrausch square root law: Λm = Λ°m − K√C.

### 3.3. Bacterial Strains and Culture Conditions

*Escherichia coli* K-12 MG1655 was used for all experiments, grown overnight at 37 °C in LB (lysogeny broth) medium (OxoidTM, Hamsphire, UK). Bacteria were maintained at −80 °C using MicrobankTM technology (Pro- Lab Diagnostics, Richmond Hill, ON, Canada).

### 3.4. Minimal Inhibitory Concentration (MIC), Growth Curves and Disk Diffusion

MIC and disk diffusion assays were performed according to the EUCAST and ISO 20776-1 guidelines [42,43] with a few modifications. MICs of the test chemicals (ILs, API-ILs and ciprofloxacin) were assessed by applying the serial two-fold dilution broth microdilution method using LB and microtiter plates. In order to create a standardized inoculum, 1 mL aliquots of the respective overnight cultures were transferred into 9 mL of fresh LB medium (1:10 dilution) and incubated for 3 h at 37 °C to ensure that cells were in a logarithmic growth phase. Subsequently, each well, which contained a serially diluted antimicrobial substance (dilution 1:2), was inoculated with 5 × 10^5^ CFU (colony-forming units) bacterial cells. After inoculation, absorbance of the 96-well microtiter plates (Corning B.V. Life Sciences, Amsterdam, The Netherlands) was measured at a wavelength of 610 nm in a TECAN F100 microplate reader (Tecan Austria GmbH, Groeding, Austria) to monitor for any possible interference by the antimicrobial substances. The microtiter plates were then incubated for 24 h at 37 °C and bacterial growth was assessed visually and confirmed by measuring the absorbances at 610 nm. The MIC was defined as the lowest concentration of the tested antimicrobial substance, at which no bacterial growth could be measured after 24 h. Results were presented as mean MICs and at least two experiments were performed on different days. Each experiment included positive (bacterial growth control in LB) and negative controls (medium without the addition of bacteria).

For growth curves, overnight cultures were diluted 1:100 and sub-cultured for approximately 2 h until OD_600nm_ reached 0.5. One hundred μl were inoculated into the wells of a microtiter plate containing 100 μL LB supplemented with serially (1:2) diluted API-ILs. Bacterial growth was monitored at OD_600nm_ in a Biotek Synergy HTX plate reader (Agilent Technologies, Santa Clara, CA, USA) at 10 min intervals for 16 h at 37 °C. Each assay was performed with five technical replicates, including positive and negative controls.

For the disk diffusion test with *E. coli*, 1 mL of an overnight culture was used to inoculate 9 mL of tryptic soy broth (TSB). This culture was grown for three hours with vigorous shaking at 37 °C to ensure that the cells were in the logarithmic growth phase. The suspension was diluted with TSB to give a respective 5 × 10^5^ CFU and immediately swabbed onto TSA plates. Up to six paper filter disks of 6 mm in diameter were aseptically placed on each plate. Afterwards, ten microliters of each IL, API-IL and ciprofloxacin dilution were applied per disk. The plates were incubated at 37 °C for 18 h. Finally, the circular zones of inhibition were measured with a ruler.

### 3.5. Mutagenesis Experiments

Mutation frequency was determined as described previously [44]. To obtain comparable results from the activity of classic antibiotics, ILs and API-ILs, the mutation frequency experiments were performed as follows. Three ml of an overnight culture *E. coli* MG1655 were diluted 1:100 in fresh LB broth and incubated for two hours at 37 °C at 200 rpm until an OD_600nm_ of 0.5 was reached. Afterwards, the subculture of *E. coli* MG1655 was again diluted 1:100 in LB medium containing ½ MIC concentration of the test compound and incubated overnight at 37 °C and 200 rpm. After incubation, the bacterial cells were pelleted via centrifugation (4.000× *g* for 10 min) and washed twice with 0.9% saline solution. After the final washing step, cells were resuspended in 10 mL fresh LB broth and incubated overnight at 37 °C and 200 rpm. After incubation, bacterial concentrations were determined by spot plating on LB agar plates and the CFU/mL values were calculated. To determine the number of mutants, 10 mL of the bacterial culture were pelleted by centrifugation, resuspended in 1 mL of 0.9% saline solution and 100 µL were plated on LB agar plates containing 100 µg/mL rifampicin. Mutation frequencies were calculated by the maximum verisimilitude method and data were processed using the online web-tool for mutation frequency determination Falcor (http://www.mitochondria.org/protocols/FALCOR.html; accessed on 3 April 2024). Each compound was tested on five different days with five independent replicates. To ensure that bacteria were able to grow under the given concentrations, growth curves were performed for each API-IL (Supplemental Figure S1) prior to mutagenesis experiments.

## 4. Conclusions

This is the first report demonstrating that changing the physico-chemical and biological properties of pharmaceuticals (in this case antibiotics) by transforming them into API-ILs can significantly influence bacterial mutation frequency, while the respective chloride ILs do not. By combining ciprofloxacin with five counter ions of different antimicrobial activities, we could demonstrate that this effect is not a general aspect of ionic liquids or even API-ILs *per se*, but is structure-dependent. Nevertheless, increased mutation frequency was found for all API-ILs demonstrating a higher mutagenesis of these compounds. At present, it is not clear whether this increased mutagenicity by API-ILs would translate into an increase in resistance evolution, and this aspect needs to be investigated further. Future studies, such as experimental evolution, can help establish whether the increased mutation frequency indeed contributes to an increased resistance to antimicrobials and whether this relationship is structure-dependent. Altogether, this will greatly contribute to a better development of API-ILs with a safer resistance profile as well as help to identify potential risks in resistance induction potential of the new IL-based antibiotics.

## Figures and Tables

**Table 1:** List of all investigated ILs, API-ILs and antibiotic including name, abbreviation, molecular weight, limiting molar conductivity and mean MIC (mg/L and mM); n.d., not determined.

Abbreviation	Name	Molecular Weight [g × mol^−1^]	A°m	MIC [mg × L^−1^]	SD	MIC [mM]
Cipro	ciprofloxacin	331.34	1.586	0.016	±0.010	0.048289
[Chol][CiP]	cholinium ciprofloxacinate	434.5	41.7	0.038	±0.007	0.087
[EMMor][CiP]	ethylmethylmorpholinium ciprofloxacinate	460.5	55.4	0.050	±0.025	0.109
[TMC_10_A][CiP]	trimethyldecylammonium ciprofloxacinate	530.7	65.1	0.050	±0.005	0.094
[TMC_16_A][CiP]	trimethylhexadecylammonium ciprofloxacinate	614.9	37.1	0.063	±0.010	0.102
[TC_8_MA][CiP]	trioctylmethylammonium ciprofloxacinate	699.0	22.4	0.050	±0.002	0.072
KCl	potassium chloride	75.6	312.2	>10,000	n.d.	>1000
[Chol][Cl]	cholinium cholride	139.6	156.5	>10,000	n.d.	>1000
[EMMor][Cl]	ethylmethylmorpholinium cholride	165.7	115.8	>10,000	n.d.	>1000
[TMC_10_A][Cl]	trimethyldecylammonium cholride	235.8	50.5	156.3	±31.3	662.8
[TMC_16_A][Cl]	trimethylhexadecylammonium cholride	320.0	83.4	23.4	±1.2	73.1
[TC_8_MA][Cl]	trioctylmethylammonium cholride	404.2	6.5	39.1	±7.4	96.7

**Table 2:** Mean inhibition zones [mm] and standard deviation of all 5 API-ILs, 5 ILs and antibiotic alone; n.d., not determined.

API-IL—Cation	Ciprofloxacin (0.5 mg/Disk)		Chloride (50 mg/Disk)	
	Mean	SD	Mean	SD
Pure antibiotic	25.00	0.0	-	
[Chol]	21.50	2.5	n.d.	
[EMMor]	20.50	0.5	n.d.	
[TMC_10_A]	20.50	3.5	8.5	0.5
[TMC_16_A]	21.00	4.0	n.d.	
[TC_8_MA]	18.50	2.5	n.d.	

## Data Availability

The original contributions presented in this study are included in the article/Supplementary Material. Further inquiries can be directed to the corresponding author(s).

## Supplementary Materials

The following supporting information can be downloaded at: https://www.mdpi.com/article/10.3390/antibiotics15060629/s1, Figure S1: Growth curves of *E. coli* MG1655 at ½ MIC of 5 API-ILs and the pure antibiotic; Table S1: Conductivity measurements of ciprofloxacin-based API-ILs and pure ciprofloxacin; Table S2: Conductivity measurements of chloride ILs; Table S3: Mean inhibition zones [mm] and standard deviation of all 5 API-ILs, 5 ILs and pure ciprofloxacin tested at different concentration per disk; Table S4: ½ MIC concentrations used for mutagenesis experiments for all API-ILs, ILs and pure ciprofloxacin investigated in this study; Table S5: Median mutation frequency including 95% confidence intervals and x-fold mutation frequency increase compared to the non-treatment controls for each of the API-ILs, ILs and pure ciprofloxacin.

## Abbreviations

The following abbreviations are used in this manuscript:

AMR	antimicrobial resistance
°C	degrees Celsius
µl	microliter
API-ILs	active pharmaceutical ingredient ionic liquids
CFU	colony-forming units
Chol	cholinium
CI	confidence intervals
ddH_2_O	double distilled water
E	exponent
EMMor	Ethylmethylmorpholinium
g	gravitational force
h	hours
HLB	hydrophilic−lipophilic balance
ILs	ionic liquids
L	liter
LB	lysogeny broth
mg	milligram
MIC	minimal inhibitory concentration
min	minutes
ml	milliliter
mM	millimolar
mm	millimeter
n.d.	not determined
nm	nanometer
OD	opitical density
QSAR	quantitative structure-activity relationship
rpm	revolutions per minute
SD	standard deviation
TC_8_MA	trioctylmethylammonium
TMC_10_A	Trimethyldecylammonium
TMC_16_A	trimethylhexadecylammonium
TSB	tryptic soy agar
WHO	world health organisation
Λ°m	limiting molar conductivity

## References

[1] Egorova K.S., Gordeev E.G., Ananikov V.P. (2017). Biological Activity of Ionic Liquids and Their Application in Pharmaceutics and Medicine. Chem. Rev..

[2] Ferraz R., Branco L.C., Prudencio C., Noronha J.P., Petrovski Z. (2011). Ionic liquids as active pharmaceutical ingredients. ChemMedChem.

[3] Hough W.L., Smiglak M., Rodriguez H., Swatloski R.P., Spear S.K., Daly D.T., Pernak J., Grisel J.E., Carliss R.D., Soutullo M.D. (2007). The third evolution of ionic liquids: Active pharmaceutical ingredients. New J. Chem..

[4] Moshikur R.M., Chowdhury M.R., Moniruzzaman M., Goto M. (2020). Biocompatible ionic liquids and their applications in pharmaceutics. Green Chem..

[5] Ferraz R., Silva D., Dias A.R., Dias V., Santos M.M., Pinheiro L., Prudêncio C., Noronha J.P., Petrovski Ž., Branco L.C. (2020). Synthesis and antibacterial activity of ionic liquids and organic salts based on penicillin g and amoxicillin hydrolysate derivatives against resistant bacteria. Pharmaceutics.

[6] Wu X., Zhu Q., Chen Z., Wu W., Lu Y., Qi J. (2021). Ionic liquids as a useful tool for tailoring active pharmaceutical ingredients. J. Control. Release.

[7] Pedro S.N., Freire C.S.R., Silvestre A.J.D., Freire M.G. (2020). The role of ionic liquids in the pharmaceutical field: An overview of relevant applications. Int. J. Mol. Sci..

[8] Ishtaweera P., Baker G.A. (2024). Progress in the application of ionic liquids and deep eutectic solvents for the separation and quantification of per- and polyfluoroalkyl substances. J. Hazard. Mater..

[9] Magina S., Barros-Timmons A., Ventura S.P.M., Evtuguin D.V. (2021). Evaluating the hazardous impact of ionic liquids—Challenges and opportunities. J. Hazard. Mater..

[10] Kemp T.J. (2012). Ionic liquids—Pharmaceutical potential. Sci. Prog..

[11] Gundolf T., Rauch B., Kalb R., Rossmanith P., Mester P. (2018). Influence of bacterial lipopolysaccharide modifications on the efficacy of antimicrobial ionic liquids. J. Mol. Liq..

[12] Guo J., Qian Y., Sun B., Sun Z., Chen Z., Mao H., Wang B., Yan F. (2019). Antibacterial Amino Acid-Based Poly(ionic liquid) Membranes: Effects of Chirality, Chemical Bonding Type, and Application for MRSA Skin Infections. ACS Appl. Bio Mater..

[13] Michalski J., Odrzygóźdź C., Mester P., Narożna D., Cłapa T. (2023). Defeat undefeatable: Ionic liquids as novel antimicrobial agents. J. Mol. Liq..

[14] Cłapa T., Michalski J., Syguda A., Narożna D., van Oostrum P., Reimhult E. (2021). Morpholinium-based ionic liquids show antimicrobial activity against clinical isolates of *Pseudomonas aeruginosa*. Res. Microbiol..

[15] Shamshina J.L., Rogers R.D. (2023). Ionic Liquids: New Forms of Active Pharmaceutical Ingredients with Unique, Tunable Properties. Chem. Rev..

[16] Qader I.B., Prasad K. (2022). Recent Developments on Ionic Liquids and Deep Eutectic Solvents for Drug Delivery Applications. Pharm. Res..

[17] Ferraz R., Branco L.C., Marrucho I.M., Araújo J.M.M., Rebelo L.P.N., Da Ponte M.N., Prudêncio C., Noronha J.P., Petrovski E. (2012). Development of novel ionic liquids based on ampicillin. Medchemcomm.

[18] Ferraz R., Teixeira V., Rodrigues D., Fernandes R., Prudêncio C., Noronha J.P., Petrovski Ž., Branco L.C. (2014). Antibacterial activity of Ionic Liquids based on ampicillin against resistant bacteria. RSC Adv..

[19] Cole M.R., Li M., El-Zahab B., Janes M.E., Hayes D., Warner I.M. (2011). Design, synthesis, and biological evaluation of β-lactam antibiotic-based imidazolium- and pyridinium-type ionic liquids. Chem. Biol. Drug Des..

[20] Araújo J.M.M., Florindo C., Pereiro A.B., Vieira N.S.M., Matias A.A., Duarte C.M.M., Rebelo L.P.N., Marrucho I.M. (2014). Cholinium-based ionic liquids with pharmaceutically active anions. RSC Adv..

[21] Mester P., Jehle A.K., Leeb C., Kalb R., Grunert T., Rossmanith P. (2016). FTIR metabolomic fingerprint reveals different modes of action exerted by active pharmaceutical ingredient based ionic liquids (API-ILs) on: *Salmonella typhimurium*. RSC Adv..

[22] Gao Y., Shu Y. (2023). Antibacterial performance of tetracycline-active drugs based on ionic liquids. J. Mol. Liq..

[23] Kumari P., Pillai V.V.S., Benedetto A. (2020). Mechanisms of action of ionic liquids on living cells: The state of the art. Biophys. Rev..

[24] Wyrzykowska E., Rybińska-Fryca A., Sosnowska A., Puzyn T. (2019). Virtual screening in the design of ionic liquids as environmentally safe bactericides. Green Chem..

[25] Rosenberg S.M., Shee C., Frisch R.L., Hastings P.J. (2012). Stress-induced mutation via DNA breaks in *Escherichia coli*: A molecular mechanism with implications for evolution and medicine. BioEssays.

[26] Kohanski M.A., DePristo M.A., Collins J.J. (2010). Sublethal Antibiotic Treatment Leads to Multidrug Resistance via Radical-Induced Mutagenesis. Mol. Cell.

[27] Pope C.F., O’Sullivan D.M., McHugh T.D., Gillespie S.H. (2008). A practical guide to measuring mutation rates in antibiotic resistance. Antimicrob. Agents Chemother..

[28] Rosche W.A., Foster P.L. (2000). Determining mutation rates in bacterial populations. Methods.

[29] Martinez J.L., Baquero F. (2000). Mutation frequencies and antibiotic resistance, Antimicrob. Agents Chemother..

[30] Rodríguez-Rojas A., Makarova O., Rolff J. (2014). Antimicrobials, Stress and Mutagenesis. PLoS Pathog..

[31] Santos M.M., Alves C., Silva J., Florindo C., Costa A., Petrovski Ž., Marrucho I.M., Pedrosa R., Branco L.C. (2020). Antimicrobial activities of highly bioavailable organic salts and ionic liquids from fluoroquinolones. Pharmaceutics.

[32] Priyanka V.P., Harikrishna A.S., Kesavan V., Gardas R.L. (2024). Synergistic interaction and antibacterial properties of surface-active mono- and di-cationic ionic liquids with ciprofloxacin. J. Mol. Liq..

[33] Maculewicz J., Świacka K., Stepnowski P., Dołżonek J., Białk-Bielińska A. (2022). Ionic liquids as potentially hazardous pollutants: Evidences of their presence in the environment and recent analytical developments. J. Hazard. Mater..

[34] Kalb R.S., Damm M., Verevkin S.P. (2017). Carbonate Based Ionic Liquid Synthesis (CBILS^®^): Development of the continuous flow method for preparation of ultra-pure ionic liquids. React. Chem. Eng..

[35] Bromberger B., Sommer J., Robben C., Trautner C., Kalb R., Rossmanith P., Mester P.-J. (2020). Evaluation of the antimicrobial activity of pyrithione-based ionic liquids. Sep. Purif. Technol..

[36] Ventura S.P.M., de Barros R.L.F., Sintra T., Soares C.M.F., Lima Á.S., Coutinho J.A.P. (2012). Simple screening method to identify toxic/non-toxic ionic liquids: Agar diffusion test adaptation. Ecotoxicol. Environ. Saf..

[37] Rodríguez-Rosado A.I., Valencia E.Y., Rodríguez-Rojas A., Costas C., Galhardo R.S., Rodríguez-Beltrán J., Blázquez J. (2019). N-acetylcysteine blocks SOS induction and mutagenesis produced by fluoroquinolones in *Escherichia coli*. J. Antimicrob. Chemother..

[38] Mohamed E.T., Wang S., Lennen R.M., Herrgård M.J., Simmons B.A., Singer S.W., Feist A.M. (2017). Generation of a platform strain for ionic liquid tolerance using adaptive laboratory evolution. Microb. Cell Fact..

[39] Reed K.B., Wagner J.M., d’Oelsnitz S., Wiggers J.M., Alper H.S. (2019). Improving ionic liquid tolerance in *Saccharomyces cerevisiae* through heterologous expression and directed evolution of an ILT1 homolog from Yarrowia lipolytica. J. Ind. Microbiol. Biotechnol..

[40] Kalb R.S., Stepurko E.N., Emel’yanenko V.N., Verevkin S.P. (2016). Carbonate based ionic liquid synthesis (CBILSregistered sign.): Thermodynamic analysis. Phys. Chem. Chem. Phys..

[41] Kalb S.W., Wesner R., Hermann W., Kotschan R.M., Schelch M. (2005). Verfahren Zur Herstllung Ionischer Flüssigkeiten, Ionischer Feststoffe Oder Gemische Derselben. Patent.

[42] The European Committee on Antimicrobial Susceptibility Testing (2026). EUCAST Disk Diffusion Method for Antimicrobial Susceptibility Testing.

[43] (2019). Clinical Laboratory Testing and In Vitro Diagnostic Test Systems—Susceptibility Testing of Infectious Agents and Evaluation of Performance of Antimicrobial Susceptibility Test Devices—Part 1: Reference Method for Testing the In Vitro Activity of Antimicrobial Agents Against Rapidly Growing Aerobic Bacteria.

[44] Rodríguez-Rojas A., Makarova O., Müller U., Rolff J. (2015). Cationic Peptides Facilitate Iron-induced Mutagenesis in Bacteria. PLoS Genet..

