# Supplementary material for: Ciprofloxacin-Based Ionic Liquids Increase Mutation Frequency in Escherichia coli
Source: Antibiotics (Basel). 2026 Jun 22;15(6):629. doi: 10.3390/antibiotics15060629 (PMC13295508; doi:10.3390/antibiotics15060629)
Supplement: Supplementary file 1 [file antibiotics-15-00629-s001.zip › antibiotics-4303761-supplementary.pdf]

Supplementary materials for the manuscript

“Ciprofloxacin-based ionic liquids increase mutation frequency in *Escherichia coli*”

by Patrick Mikuni-Mester, Birgit Bromberger, Timea Dömök, Daniela Zetner, Laura Schleifer and Olga Makarova

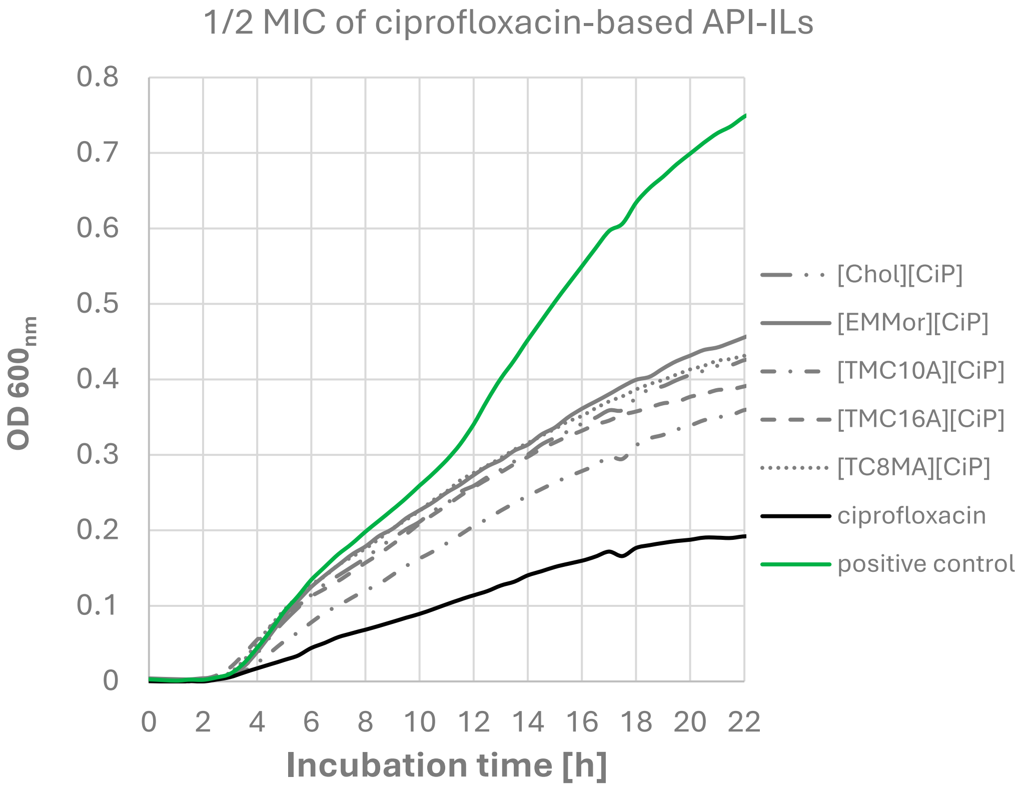

Figure S1: Growth curves of *E. coli* MG1655 at ½ MIC of 5 API-ILs and the pure antibiotic.

Table S1: Conductivity measurements of ciprofloxacin-based API-ILs and pure ciprofloxacin.

	Concentration (mol)	√C	Conductivity (mS)	Am		Concentration (mol)	√C	Conductivity (mS)	Am
	0.0250	0.16	0.007	0.3		0.0250	0.16	0.687	27.5
	0.0167	0.13	0.005	0.3		0.0167	0.13	0.620	37.2
	0.0125	0.11	0.005	0.4		0.0125	0.11	0.509	40.7
	0.0083	0.09	0.004	0.4		0.0083	0.09	0.383	46.0
	0.0063	0.08	0.004	0.6		0.0063	0.08	0.303	48.5
	0.0050	0.07	0.003	0.7		0.0050	0.07	0.235	47.0
**Cipro**	0.0042	0.06	0.003	0.8	**[TMC$_{10}$A] [CiP]**	0.0042	0.06	0.209	50.2
	0.0036	0.06	0.003	0.8		0.0036	0.06	0.183	51.2
	0.0031	0.06	0.003	0.9		0.0031	0.06	0.165	52.7
	0.0025	0.05	0.003	1.1		0.0025	0.05	0.137	54.6
	0.0021	0.05	0.003	1.2		0.0021	0.05	0.107	51.4
	0.0018	0.04	0.002	1.4		0.0018	0.04	0.100	56.1
	0.0016	0.04	0.003	1.7		0.0016	0.04	0.090	57.5
	0.0250	0.16	0.638	25.5		0.0250	0.16	0.174	6.9
	0.0167	0.13	0.626	37.6		0.0167	0.13	0.187	11.2
	0.0125	0.11	0.459	36.7		0.0125	0.11	0.117	9.3
	0.0083	0.09	0.320	38.4		0.0083	0.09	0.155	18.6
	0.0063	0.08	0.246	39.4		0.0063	0.08	0.120	19.3
	0.0050	0.07	0.190	38.0		0.0050	0.07	0.100	20.0
**[Chol] [CiP]**	0.0042	0.06	0.159	38.2	**[TMC$_{16}$A] [CiP]**	0.0042	0.06	0.087	20.9
	0.0036	0.06	0.127	35.7		0.0036	0.06	0.079	22.1
	0.0031	0.06	0.120	38.5		0.0031	0.06	0.075	23.9
	0.0025	0.05	0.093	37.1		0.0025	0.05	0.063	25.3
	0.0021	0.05	0.080	38.2		0.0021	0.05	0.058	27.7
	0.0018	0.04	0.066	37.2		0.0018	0.04	0.052	28.9
	0.0016	0.04	0.060	38.4		0.0016	0.04	0.048	31.0
	0.0250	0.16	1.071	42.8		0.0250	0.16	0.148	5.9
	0.0167	0.13	0.982	58.9		0.0167	0.13	0.149	8.9
	0.0125	0.11	0.726	58.1		0.0125	0.11	0.109	8.8
	0.0083	0.09	0.462	55.4		0.0083	0.09	0.106	12.7
	0.0063	0.08	0.342	54.7		0.0063	0.08	0.071	11.3
	0.0050	0.07	0.267	53.4		0.0050	0.07	0.059	11.8
**[EMMor] [CiP]**	0.0042	0.06	0.220	52.8	**[TC$_8$MA] [CiP]**	0.0042	0.06	0.062	15.0
	0.0036	0.06	0.194	54.3		0.0036	0.06	0.059	16.6
	0.0031	0.06	0.167	53.4		0.0031	0.06	0.053	16.9
	0.0025	0.05	0.134	53.6		0.0025	0.05	0.044	17.5
	0.0021	0.05	0.111	53.2		0.0021	0.05	0.037	17.7
	0.0018	0.04	0.095	53.4		0.0018	0.04	0.033	18.4
	0.0016	0.04	0.083	53.0		0.0016	0.04	0.030	18.9

Table S2: Conductivity measurements of chloride ILs.

	Concentration (mol)	√C	Conductivity (mS)	Am		Concentration (mol)	√C	Conductivity (mS)	Am
**KCl**	0.0250	0.16	3.320	132.8	**[TMC$_{10}$A] [Cl]**	0.0250	0.16	0.567	22.7
	0.0167	0.13	2.650	159.0		0.0167	0.13	0.717	43.0
	0.0125	0.11	2.330	186.4		0.0125	0.11	0.549	43.9
	0.0083	0.09	1.074	128.9		0.0083	0.09	0.384	46.1
	0.0063	0.08	1.416	226.6		0.0063	0.08	0.287	45.9
	0.0050	0.07	1.197	239.4		0.0050	0.07	0.241	48.2
	0.0042	0.06	1.024	245.8		0.0042	0.06	0.185	44.4
	0.0036	0.06	0.888	248.6		0.0036	0.06	0.163	45.7
	0.0031	0.06	0.777	248.6		0.0031	0.06	0.152	48.6
	0.0025	0.05	0.639	255.6		0.0025	0.05	0.124	49.6
	0.0021	0.05	0.538	258.2		0.0021	0.05	0.092	43.9
	0.0018	0.04	0.465	260.4		0.0018	0.04	0.089	49.6
	0.0016	0.04	0.407	260.5		0.0016	0.04	0.077	49.1
**[Chol] [Cl]**	0.0250	0.16	2.990	119.6	**[TMC$_{16}$A] [Cl]**	0.0250	0.16	0.469	18.8
	0.0167	0.13	1.988	119.3		0.0167	0.13	0.716	43.0
	0.0125	0.11	1.536	122.9		0.0125	0.11	0.528	42.2
	0.0083	0.09	1.255	150.6		0.0083	0.09	0.462	55.4
	0.0063	0.08	0.915	146.4		0.0063	0.08	0.349	55.8
	0.0050	0.07	0.729	145.8		0.0050	0.07	0.301	60.2
	0.0042	0.06	0.635	152.4		0.0042	0.06	0.257	61.7
	0.0036	0.06	0.534	149.5		0.0036	0.06	0.241	67.5
	0.0031	0.06	0.477	152.6		0.0031	0.06	0.210	67.2
	0.0025	0.05	0.369	147.6		0.0025	0.05	0.175	69.8
	0.0021	0.05	0.315	151.2		0.0021	0.05	0.141	67.5
	0.0018	0.04	0.268	150.1		0.0018	0.04	0.123	69.1
	0.0016	0.04	0.233	149.1		0.0016	0.04	0.103	66.1
**[EMMor] [Cl]**	0.0250	0.16	2.080	83.2	**[TC$_8$MA] [Cl]**	0.0250	0.16	0.056	2.2
	0.0167	0.13	1.634	98.0		0.0167	0.13	0.038	2.3
	0.0125	0.11	1.269	101.5		0.0125	0.11	0.082	6.6
	0.0083	0.09	0.871	104.5		0.0083	0.09	0.056	6.8
	0.0063	0.08	0.667	106.7		0.0063	0.08	0.042	6.7
	0.0050	0.07	0.531	106.2		0.0050	0.07	0.037	7.3
	0.0042	0.06	0.445	106.8		0.0042	0.06	0.030	7.2
	0.0036	0.06	0.380	106.4		0.0036	0.06	0.021	5.9
	0.0031	0.06	0.335	107.2		0.0031	0.06	0.020	6.4
	0.0025	0.05	0.270	108.0		0.0025	0.05	0.018	7.2
	0.0021	0.05	0.221	106.1		0.0021	0.05	0.014	6.7
	0.0018	0.04	0.188	105.2		0.0018	0.04	0.011	6.3
	0.0016	0.04	0.167	106.8		0.0016	0.04	0.010	6.4

Table S3: Mean inhibition zones [mm] and standard deviation of all 5 API-ILs, 5 ILs and pure ciprofloxacin tested at different concentration per disc.

	50 mg/disc		25 mg/disc		10 mg/disc	
	Inhibition zone [mm]					
	**Mean**	**SD**	**Mean**	**SD**	**Mean**	**SD**
Cipro	29.0	0.0	25.0	0.0	18.5	0.5
[Chol][CiP]	26.0	3.0	21.5	2.5	14.0	3.0
[EMMor][CiP]	26.0	0.0	20.5	0.5	13.5	1.5
[TMC$_{10}$A][CiP]	26.0	2.0	20.5	3.5	12.0	4.0
[TMC$_{16}$A][CiP]	26.5	2.5	21.0	4.0	15.0	4.0
[TC$_8$MA][CiP]	27.0	2.0	18.5	2.5	12.0	3.0
[Chol][Cl]	n.d.	n.d.	n.d.	n.d.	n.d.	n.d.
[EMMor][Cl]	n.d.	n.d.	n.d.	n.d.	n.d.	n.d.
[TMC$_{10}$A][Cl]	18.0	3.0	13.0	1.0	8.5	0.5
[TMC$_{16}$A][Cl]	n.d.	n.d.	n.d.	n.d.	n.d.	n.d.
[TC$_8$MA][Cl]	10.0	1.0	9.0	1.0	9.0	0.0

Table S4: ½ MIC concentrations used for mutagenesis experiments for all API-ILs, ILs and pure ciprofloxacin investigated in this study.

API-IL - cation	1/2 MIC concentration used for Mutagensis experiments [mg/L]	
	**Cirofloxacin**	**Chloride**
Pure antibiotic	0,01	
[Chol]	0,02	5,0
[EMMor]	0,03	15,0
[$TMC_{10}A$]	0,03	100,0
[$TMC_{16}A$]	0,03	10,0
[TC_8MA]	0,03	10,0

Table S5: Median mutation frequency including 95% confidence intervals and x-fold mutation frequency increase compared to the non-treatment controls for each of the API-ILs, ILs and pure ciprofloxacin.

		Median Mutation frequency	Upper Bound 95% CI Range	Lower Bound 95% CI Range	Upper Difference 95% CI Median +/-	Lower Difference 95% CI Median +/-	x-fold increase compared to non-treatment control
**n = 25** 5 technical replicates on 5 different days	Non-treatment	1,46E-09	2,79E-09	7,00E-10	1,33E-09	7,60E-10	1,00
	Ciprofloxacin	2,50E-09	6,75E-09	1,52E-09	4,25E-09	9,80E-10	1,71
	[Chol][CiP]	5,97E-09	9,09E-09	2,19E-09	3,12E-09	3,78E-09	4,09
	[EMMor][CiP]	3,79E-09	5,59E-09	2,25E-09	1,80E-09	1,54E-09	2,60
	[$TMC_{10}A$][CiP]	1,02E-08	1,56E-08	5,61E-09	5,41E-09	4,60E-09	6,99
	[$TMC_{16}A$][CiP]	7,33E-09	1,32E-08	3,14E-09	5,84E-09	4,19E-09	5,02
	[TC_8MA][CiP]	8,62E-09	1,72E-08	4,76E-09	8,54E-09	3,86E-09	5,90
**n = 25** 5 technical replicates on 5 different days	LB	2,86E-09	3,50E-09	1,98E-09	6,40E-10	8,80E-10	1,00
	[Chol][Cl]	3,45E-09	8,30E-09	2,20E-09	4,85E-09	1,25E-09	1,21
	[EMMor][Cl]	2,38E-09	4,14E-09	1,63E-09	1,76E-09	7,50E-10	0,83
	[TMC10A][Cl]	4,71E-09	6,39E-09	2,53E-09	1,68E-09	2,18E-09	1,65
	[TMC16A][Cl]	1,77E-09	2,59E-09	1,40E-08	8,20E-10	3,70E-10	0,62
	[TC_8MA][Cl]	2,35E-09	3,73E-09	1,49E-09	1,38E-09	8,60E-10	0,82
